# Supplementary material for: Inflammation Differentially Modulates the Biological Features of Adult Derived Human Liver Stem/Progenitor Cells
Source: Cells. 2020 Jul 8;9(7):1640. doi: 10.3390/cells9071640 (PMC7408415; doi:10.3390/cells9071640)
Supplement: Supplementary file 1 [file cells-09-01640-s001.pdf]

SUPPLEMENTARY MATERIAL

Supplementary figure 1: Effect of inflammation on cytokine and cytokine receptors gene expression

Box plot presenting the differentially expressed cytokines and cytokines receptors in ADHLSC primed with inflammatory cocktail for 24h and 9 days. For each condition (24h and 9 days), the difference in gene expression, as compared to control, is presented by Δct (Ct gene - Ct housekeeping gene) of each significantly expressed gene after normalization (see materials and methods) (n=3) $p<0.01$. *A*, plots of 15 upregulated and 5 down regulated genes in ADHLSC after 24h of inflammation priming. *B*, plots of 19 upregulated and 3 down regulated genes after 9 days of inflammation priming among the 96 cytokines and cytokines receptors analyzed.

El-Kehdy et al, Supplementary Figure 1

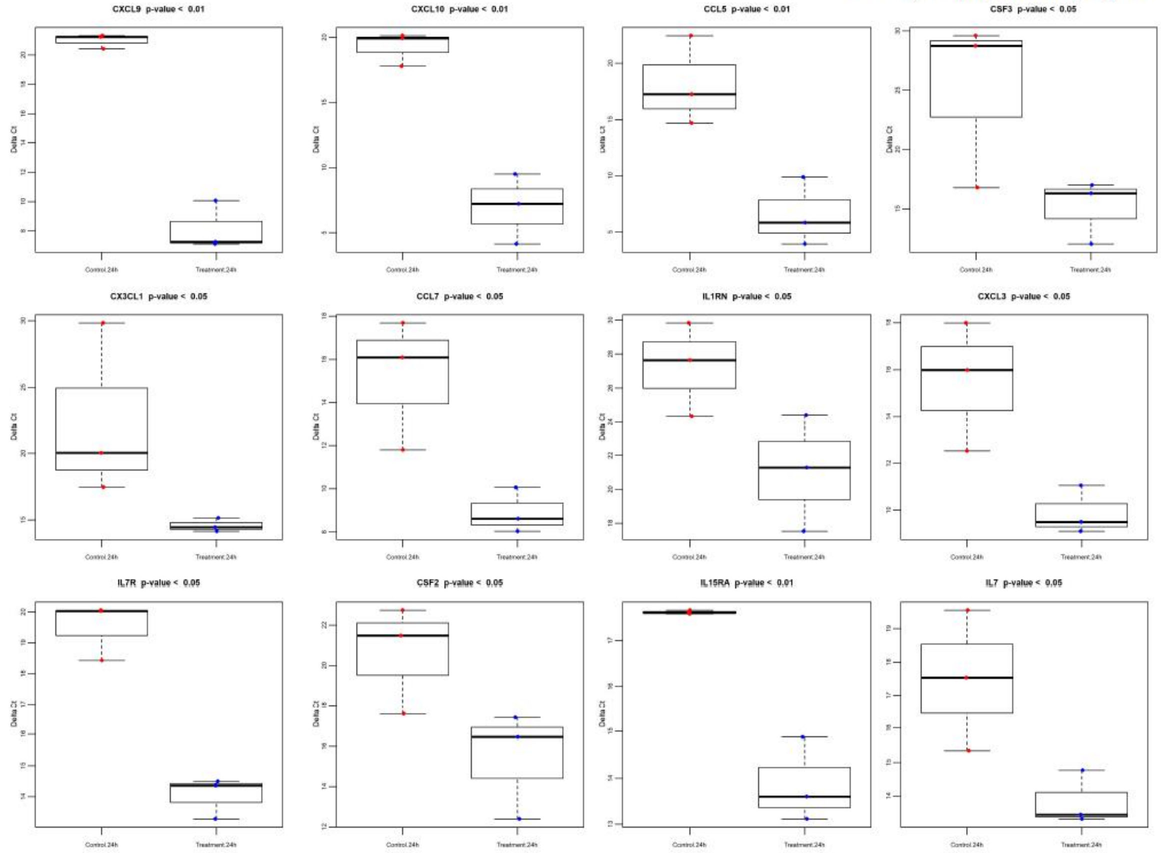

El-Kehdy et al, Supplementary Figure 1

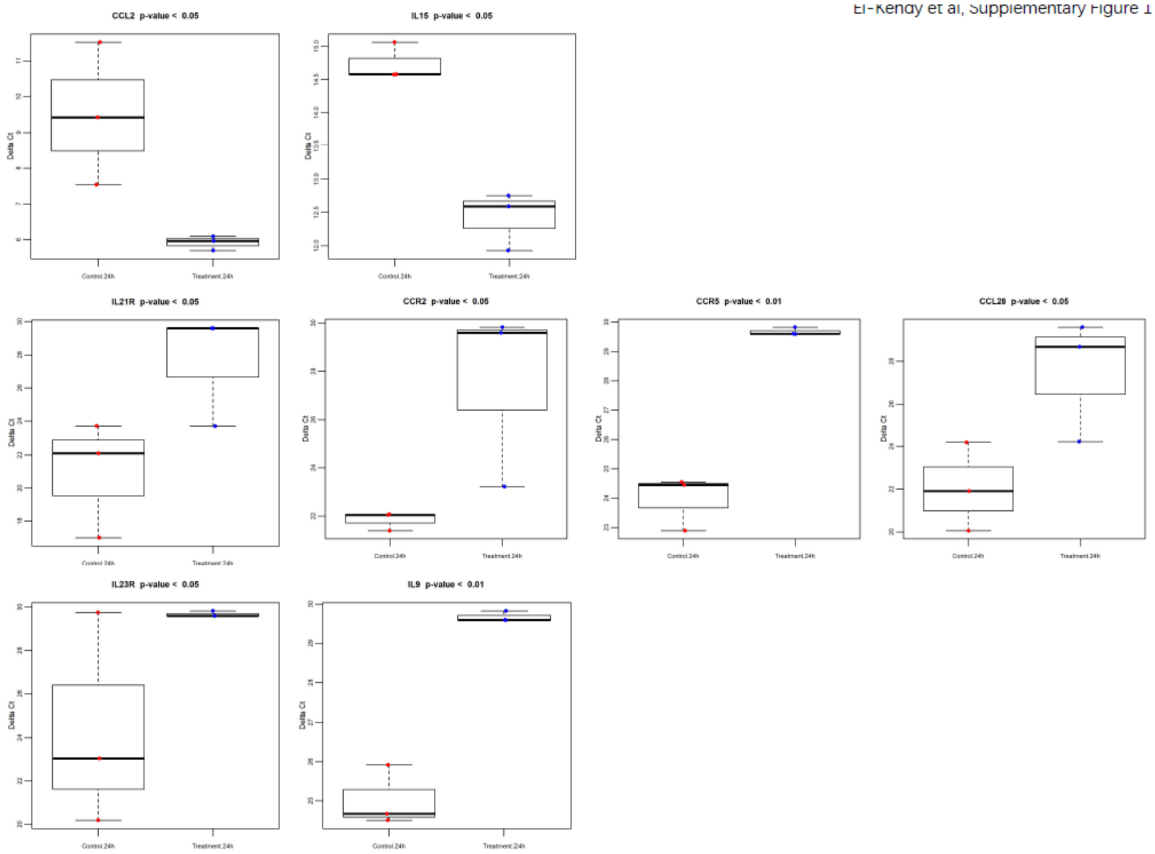

El-Kehdy et al. Supplementary Figure 1

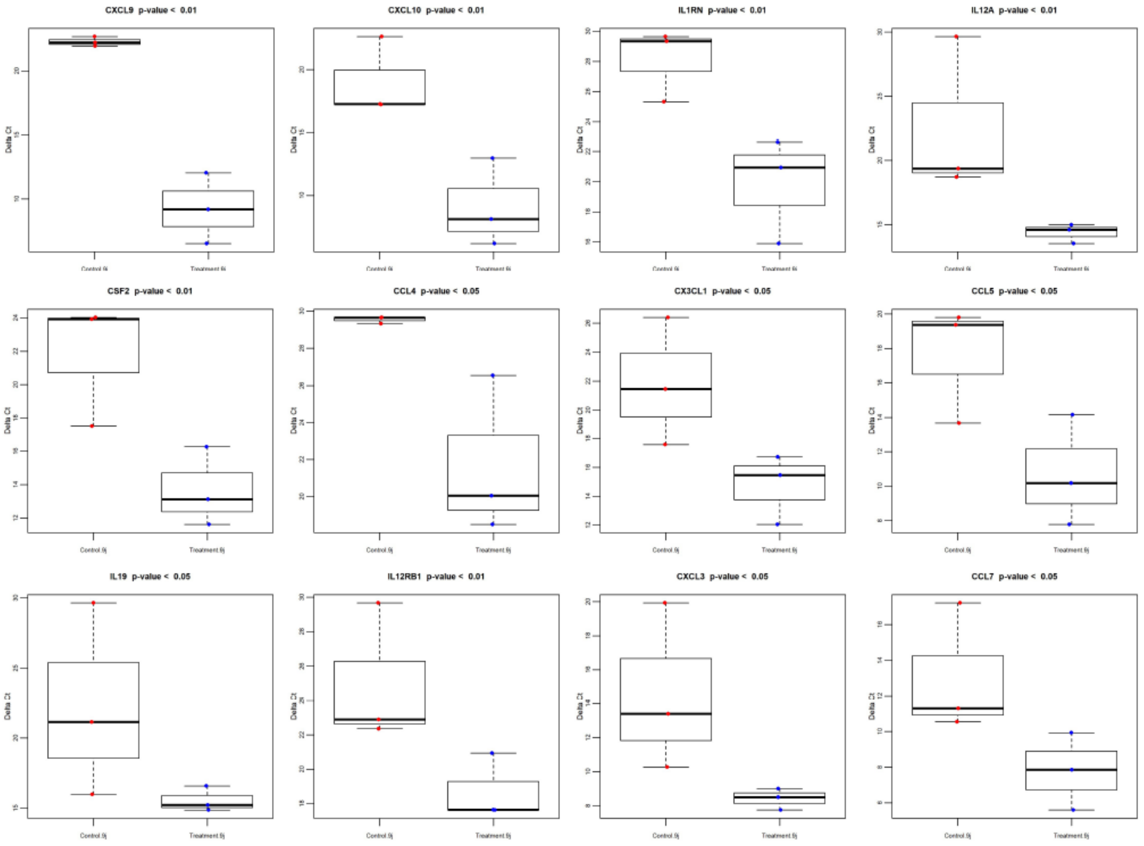

El-Kehdy et al, Supplementary Figure 1

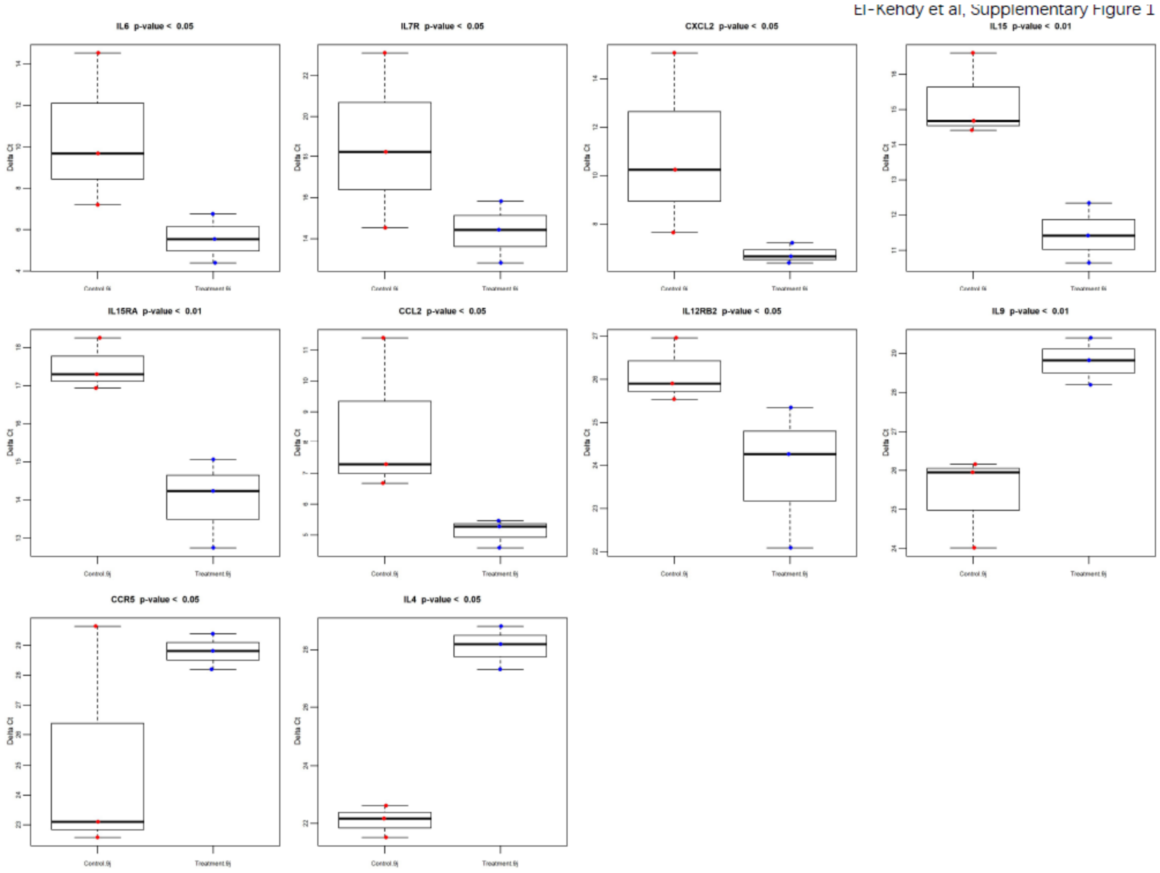

Supplementary figure 2: Effect of 30 days-inflammation on ADHLSC hepatogenic differentiation potential.

A, alteration of the typical morphological changes noted after in vitro hepatogenic differentiation of ADHLSC when the inflammation cocktail is added. Magnification: 200 x

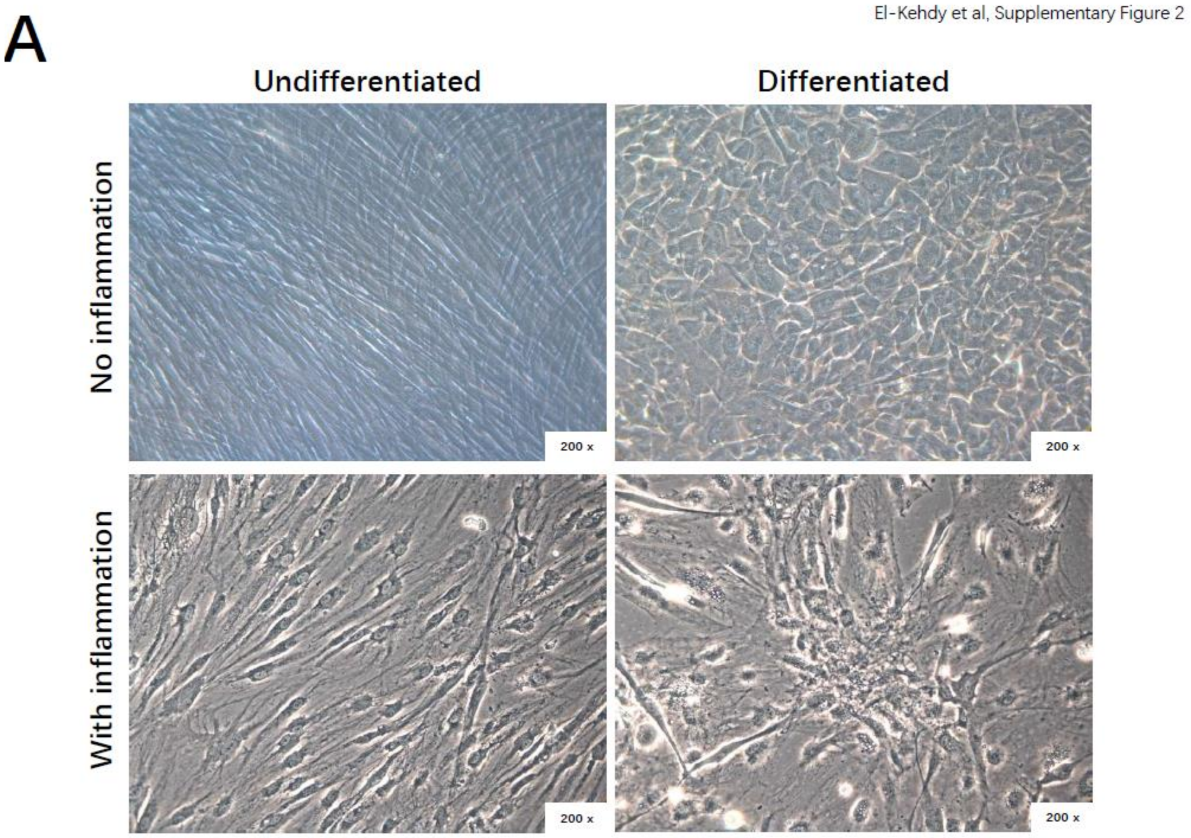

B & C, RT-qPCR gene expression analysis demonstrated the inflammation does not alter the mRNA expression of mesenchymal markers expect for slug which expression

remains upregulated. However, inflammation significantly inhibits the upregulation of hepatocytic markers that normally occurs after hepatogenic differentiation which is in correlation with the morphological changes. For treated and untreated groups, results are expressed as fold change in differentiated versus corresponding undifferentiated ADHLSC.

CD54 (Intercellular Adhesion Molecule 1; ICAM-1), Sox9 (SRY-Related HMG-Box 9 encoding gene), Snail (SNAI1), Slug (SNAI2), VIM (Vimentin), MRP2 (Multi-drug resistance-associated protein-2 encoding gene), TAT (Tyrosine aminotransferase), FAH (Fumarylacetoacetate hydrolase), ALB (Albumin).

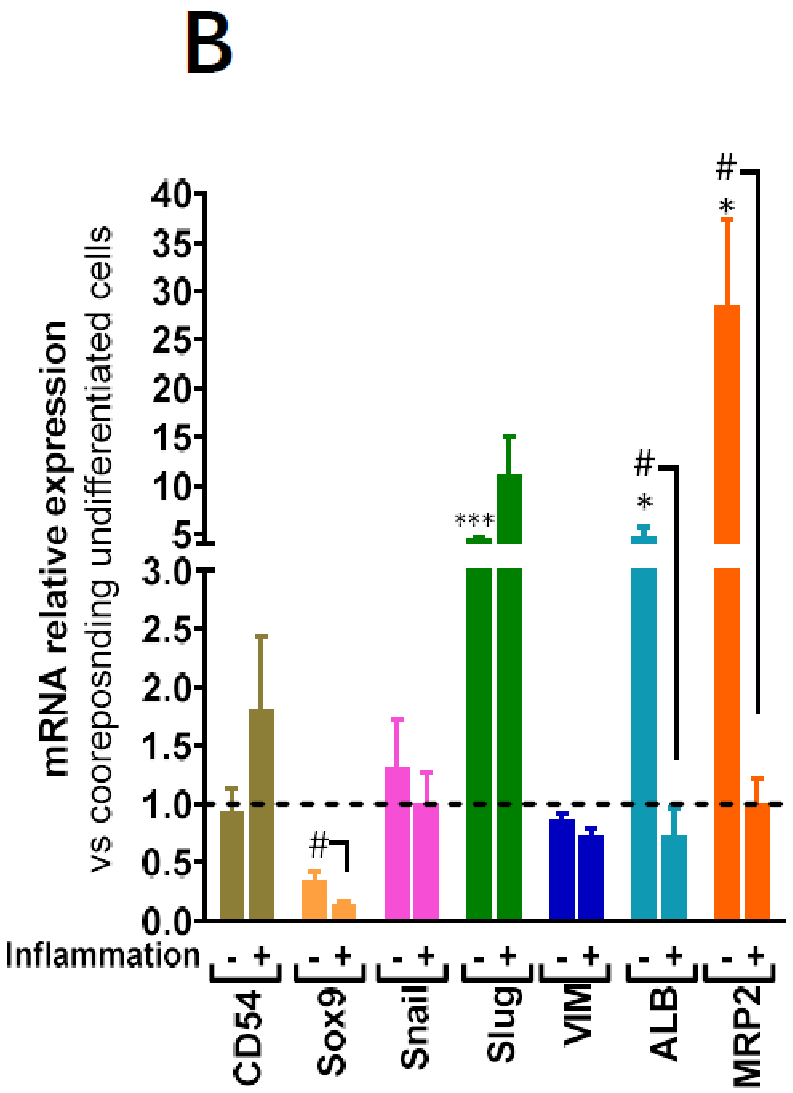

C: Undifferentiated and differentiated ADHLSC from untreated and treated groups were incubated with IPA substrate and luciferase activity was measured. Results are expressed as the relative luminescence unit detected in the differentiated ADHLSC versus undifferentiated counterparts. Data shown are the mean ± SEM of at least 4 independent experiments.

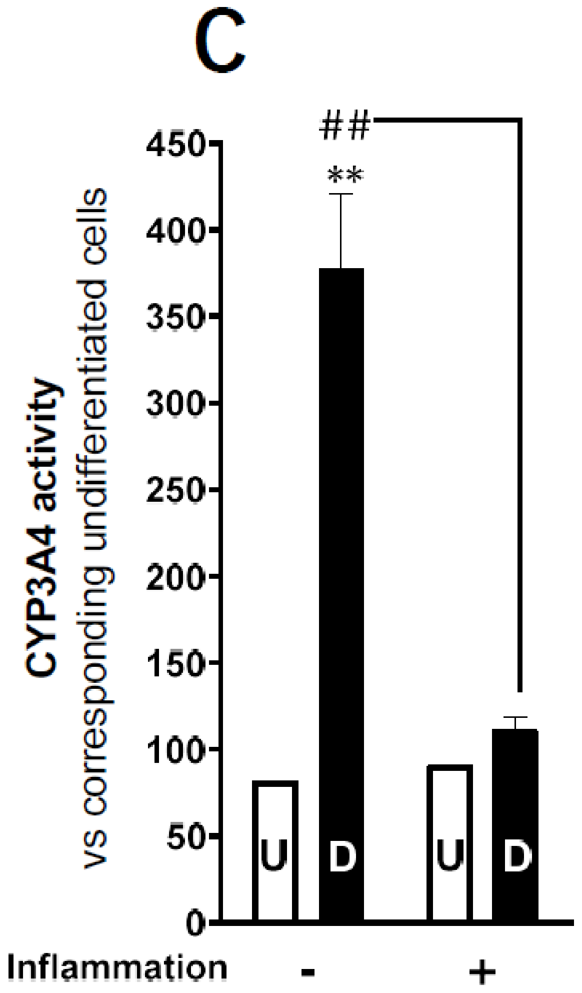
C
CYP3A4 activity
vs corresponding undifferentiated cells
450
400
350
300
250
200
150
100
50
0
##
**
U
D
U
D
Inflammation
-
+
